# Transcriptomic profiling of human breast and melanoma cells selected by migration through narrow constraints

**DOI:** 10.1038/sdata.2017.172

**Published:** 2017-11-14

**Authors:** Dominika A. Rudzka, William Clark, Ann Hedley, Gabriela Kalna, Michael F. Olson

**Affiliations:** 1 Cancer Research UK Beatson Institute, Garscube Estate, Switchback Road, Glasgow G61 1BD, UK; 2 Institute of Cancer Sciences, University of Glasgow, Glasgow G12 8QQ, UK

**Keywords:** Metastasis, Cell invasion

## Abstract

The metastatic spread of cancer cells is a step-wise process that starts with dissociation from primary tumours and local invasion of adjacent tissues. The ability to invade local tissues is the product of several processes, including degradation of extracellular matrices (ECM) and movement of tumour cells through physically-restricting gaps. To identify properties contributing to tumour cells squeezing through narrow gaps, invasive MDA-MB-231 human breast cancer and MDA-MB-435 human melanoma cells were subjected to three successive rounds of selection using cell culture inserts with highly constraining 3 μm pores. For comparison purposes, flow cytometry was also employed to enrich for small diameter MDA-MB-231 cells. RNA-Sequencing (RNA-seq) using the Illumina NextSeq 500 platform was undertaken to characterize how gene expression differed between parental, invasive pore selected or small diameter cells. Gene expression results obtained by RNA-seq were validated by comparing with RT-qPCR. Transcriptomic data generated could be used to determine how alterations that enable cell passage through narrow spaces contribute to local invasion and metastasis.

## Background & Summary

The metastatic spread of cancer cells from primary tumours to distant sites is the most serious and deadliest aspect of the disease, with estimates of up to 90% of cancer related deaths being directly associated with metastasis^1,2^. In addition, it has become apparent that the same processes that contribute to cancer metastasis also facilitate primary tumour growth^3,4^. As a result, characterizing the properties of metastatic cells that enable their dissemination may identify actionable targets for chemotherapy that could reduce cancer spread and possibly tumour growth and progression.

The metastatic ability of cancer cells results from several changes in their normal behaviours^5^. The strength of cell-cell adhesions is often lessened, allowing individual or groups of cells to separate and move away from the primary tumour^6^. The movement away from the tumour into adjacent tissue may be promoted by changes in migratory behaviour, increased extracellular matrix (ECM) degrading activity, and the ability to squeeze between cells and ECM protein fibres^7^. In the next stage, locally invasive cells may spread further by moving through surfaces surrounding body cavities, via lymphatic or blood vessels, or through canalicular spaces. Ultimately, tumour cells may move to a secondary site, the location of which may be influenced by variables including the route taken, intrinsic properties of the target tissue, and accessibility to factors, such as tumour cell-generated exosomes, that condition a pre-metastatic niche. Common to several stages of the metastatic process is the ability of tumour cells to squeeze through narrow spaces. As a result, it can be predicted that changes in the deformability of tumour cells that enabled their movement through physically constraining conditions in their three dimensional environment would likely promote cancer spread^8^.

**Figure 1:**
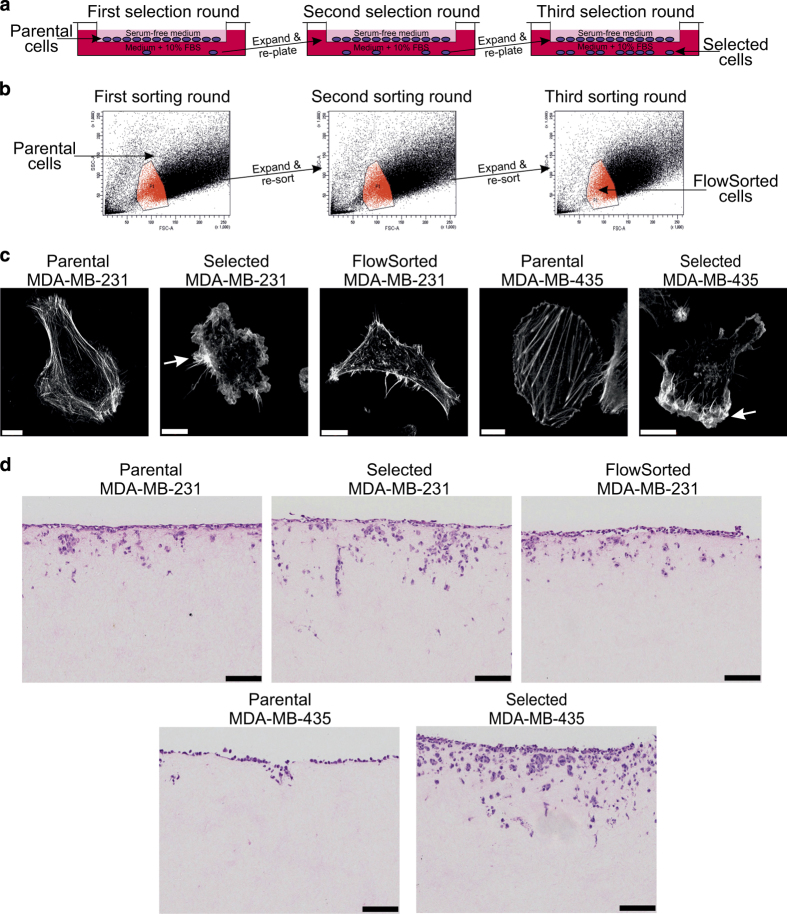
Selection strategies to enrich for invasive or small diameter cells. (**a**) MDA-MB-231 or MDA-MB-435 cells were plated in serum-free medium into tissue culture inserts with 3 μm pores, and placed in tissue culture dishes containing medium with 10% fetal bovine serum (FBS). Cells able to migrate through pores and attach to the underlying dishes were expanded, re-plated and the selection repeated twice more. At the end, several independent Selected cell populations were established. (**b**) MDA-MB-231 cells were sorted by flow cytometry (side scatter and forward scatter) and gated in the P1 region (red) for small diameter cells. After two additional rounds of expansion and sorting, several independent FlowSorted populations were established. (**c**) Representative Parental, Selected and FlowSorted MDA-MB-231 cells, as well as Parental and Selected MDA-MB-435 cells, were fixed, stained with Alexa Fluor488-conjugated phalloidin to enable visualization of filamentous actin structures. Actin-rich protrusive regions have been indicated for Selected cells with white arrows. Scale bars=10 μm. (**d**) H&E‐stained sections of cell invasion into collagen matrix after 8 days. Scale bar=100 μm.

To select for cells that were better able to move through narrow gaps, MDA-MB-231 D3H2LN human breast cancer cells expressing firefly luciferase (Luc)^9^ (abbreviated MDA-MB-231) and MDA-MB-435 human melanoma cells (which were mistakenly used in the past as a breast cancer model until its cancer type was corrected)^10^ were subjected to three rounds of enrichment using tissue culture inserts with 3 μm pores (Fig. 1a). By plating cells on microporous membranes in serum-free medium in the inserts, and then transferring the inserts to tissue cultures dishes containing medium supplemented with 10% fetal bovine serum (FBS), a chemotactic gradient was created to attract cells to move through the restricting narrow pores. Several independent ‘Selected’ populations of cells were established from both MDA-MB-231 and MDA-MB-435 cells in this manner. Given the possibility that selection through narrow pores would enrich for small cells, several independent ‘FlowSorted’ populations of small diameter MDA-MB-231 cells were also selected by three consecutive rounds of flow cytometry (Fig. 1b).

Parental, Selected and FlowSorted populations of MDA-MB-231 and MDA-MB-435 cells were fixed and stained with phalloidin to reveal filamentous actin (F-actin) structures and cell morphology. Selected and FlowSorted MDA-MB-231 cells, as well as Selected MDA-MB-435 cells, were notably smaller than their originating parental cells (Fig. 1c). In addition, Selected cells from both tumour cell lines were marked by fewer cytoplasmic and cortical distinct F- actin fibres, more actin-rich protrusive regions (indicated by white arrows), and more irregular circumferential membranes compared to their respective parents (Fig. 1c). When invasion into three dimensional fibroblast-conditioned dense collagen matrices was examined, Selected MDA-MB-231cells were markedly more invasive than Parental or FlowSorted cells (Fig. 1d). In addition, pore-selection changed relatively non-invasive Parental MDA-MB-435 cells into highly invasive Selected cells (Fig. 1d).

**Figure 2:**
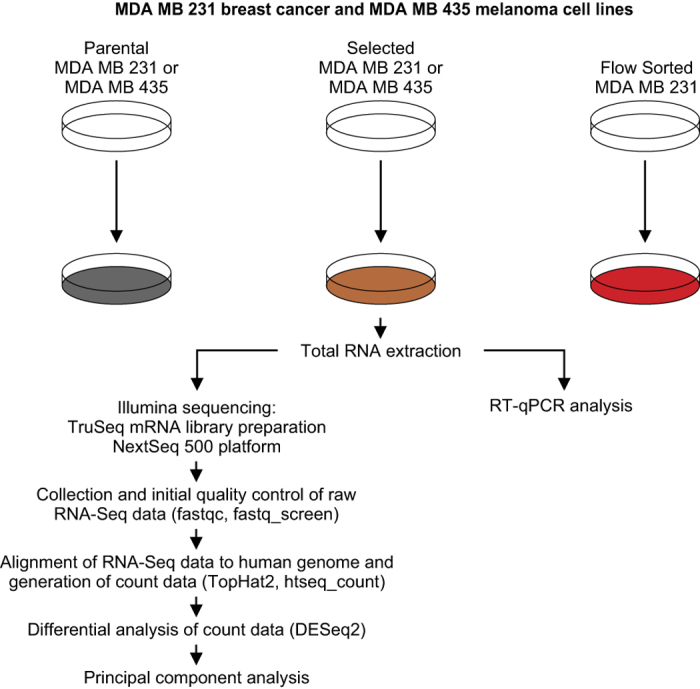
Overview and experimental design of the study.

To determine how gene expression differs between Parental, Selected and FlowSorted populations of MDA-MB-231 and MDA-MB-435, total RNA was extracted and enriched for polyA+ mRNAs, and then subjected to RNA-Sequencing (Fig. 2 and Table 1). The study has been described at the NCBI Bioproject (PRJNA327913), with descriptions of the MDA-MB-231 cells (SAMN07311741) and MDA-MB-435 cells (SAMN07311743). Primary data are available at the NCBI Sequence Read Archive (Table 2 (available online only) and Data Citation 1). The transcriptomic data generated by this study may reveal important modifiers of the physical properties that enable tumour cells to move through narrow spaces, as well as regulators of cell size, that contribute to the metastatic spread of cancer.

## Methods

### Cell culture

Human MDA-MB-231 D3H2LN Luc^9^ (abbreviated MDA-MB-231) breast cancer cells (Caliper LifeScience, Hopkinton MA USA) and MDA-MB-435 melanoma cells (ATCC, Teddington UK) were grown in Hyclone MEM/EBSS media, supplemented with 10% fetal bovine serum (FBS), 2 mM L-glutamine, 10 U/ml penicillin and 10 μgml^−1^ streptomycin, 1% MEM/NEAA (non-essential amino acid), 1% Sodium Pyruvate (all Gibco, Fisher Scientific, Loughborough, UK) at 37 °C with 5% CO_2_ in a humidified incubator. Cell line identities were validated by the Cancer Research UK Beatson Institute Molecular Services using the GenePrint 10 system STR multiplex assay (Promega, Southampton UK) that amplifies 9 tetranucleotide repeat loci and Amelogenin gender determining marker.

### Cell line selection

Independent MDA-MB-231 or MDA-MB-435 pore-selected (Selected) cell populations were established by seeding 1×10^6^ cells in 10 ml serum-free medium on 3 μm pore membranes in 7.5 cm cell culture inserts (Corning, Fisher Scientific, Loughborough, UK). Inserts were placed in 10 cm dishes containing 10 ml serum-containing medium, and left for four days in standard tissue culture conditions to allow cells to migrate through the pores. The inserts were then removed, media was changed and plates were placed back in the incubator to expand the selected cell population. The selection process was repeated twice more as described above.

Independent small diameter MDA-MB-231 flow cytometry sorted (FlowSorted) populations were obtained by gating on cells with low forward scatter and side scatter parameters as indicated in the red region in Fig. 1b using a FACSAria Fusion sorter (BD Biosciences, Oxford UK). FlowSorted cells were then grown using standard tissue culture conditions to expand the isolated sorted cell populations, followed by two additional rounds of sorting as described above.

Cells were stained and imaged as described in ref. 11. For filamentous actin imaging, 0.8×10^5^ cells were seeded on 13 mm autoclaved cover slips and grown overnight. Cells were washed with phosphate buffered saline (PBS), then fixed with 4% paraformaldehyde/PBS solution, permeabilized with 0.5% (v/v) Triton X-100/PBS, blocked with 1% (w/v) bovine serine albumin (Sigma-Aldrich, Gillingham, UK)/PBS, and then incubated with Alexa Fluor488 phalloidin (Sigma-Aldrich, Gillingham, UK) (1:1,000 dilution) for 1 h at room temperature. Cover slips were washed twice with PBS and inverted on 7 μl of ProLong Diamond Antifade Mountant with DAPI (Thermo Fisher Scientific, Renfrew UK) on a glass slide. Cells were imaged on a Zeiss 880 confocal microscope (Cambridge UK) using a 63X oil objective.

Collagen matrix invasion assays were performed as previously described^11^. Primary human fibroblasts were mixed with rat tail collagen1 and placed in a cell culture incubator for a week to allow conditioning of the collagen. To remove fibroblasts from the collagen matrices, the disks were incubated with 5 μgml^−1^ Puromycin for at least 24 h and then washed twice with medium. 2×10^5^ cells were seeded on top of the disks and allowed to settle and grow over 2 days. Afterward, the collagen matrices were mounted onto grids to generate an air/liquid interface. After 8 days, the collagen disks were fixed in 4% paraformaldehyde overnight and processed using standard histological methods. H&E‐stained sections were scanned and analyzed using Digital Slide Server (SlidePath, Leica, Milton Keynes UK) software.

### RNA isolation

1×10^6^ cells were seeded into 6-well plates and allowed to settle and grow overnight. Cells were harvested with Trypsin and total RNA was extracted using the RNAeasy kit (Qiagen, Manchester UK) according to manufacturer’s instructions. RNA was quantified using the Nanodrop spectrophotometer (Nanodrop, Thermo Fisher Scientific, Renfrew UK). The Agilent RNA ScreenTape assay and the Agilent 2,200 TapeStation system (both Agilent, Stockport UK) were used to determine the RNA integrity number equivalent (RINe; Table 3).

### RNA-sequencing

Total RNA was used to generate an oligo dT-enriched library with the Illumina TruSeq RNA Library Preparation kit v2.0 (Illumina, Cambridge UK). Quality and quantity of the DNA library was assessed using the Agilent 2,100 Bioanalyzer (Agilent, Stockport UK) and the Qubit (Thermo Fisher Scientific, Renfrew UK), respectively. The library was run on the Illumina NextSeq 500 platform using the High Output 75 cycles kit (2×36 cycles, paired-end reads, single index) (both Illumina, Cambridge UK).

### RNA-sequence analysis

RNA-Sequence analysis and alignment was carried out as described in reference^12^. Quality control checks of raw RNA-Seq data files were done with fastqc v0.10.1 (http://www.bioinformatics.babraham.ac.uk/projects/fastqc/) and fastq_screen v0.4.2 (http://www.bioinformatics.babraham.ac.uk/projects/fastq_screen/). RNA-Seq reads were aligned to the human genome build GRCh38 with TopHat2.0.13^13^ and genome annotation using GRCh38.82.gtf. BAM files were further processed with HTseq0.6.1p1 (http://www.huber.embl.de/users/anders/HTSeq/doc/count.html). Differential analysis of count data was performed by the DESeq2 package (DESeq2)^14^. Regularized log transformation was used to transform the DESeq2 data for principal component analysis.

### Quantitative PCR

Total RNA was used to prepare complementary DNA (cDNA) using a Quantitect Reverse Transcription kit (Qiagen, Manchester UK). To perform quantitative PCR, the DyNAmo HS SYBR Green qPCR Kit (Thermo Fisher Scientific, Renfrew UK) was used in triplicate in a 20 μl reaction mixture containing 10 μl of master mix (master mix contains hot-start polymerase, SYBR green, PCR buffer, 5 mM MgCl_2_, and dNTP mix), 6.35 μl of nuclease-free water, 0.15 μl of primer, and 0.4 μl ROX passive reference dye. Primer catalogue numbers (Qiagen, Manchester UK) are in Table 4. Reaction mixtures were distributed into MicroAmp Fast Optical 96-well plates and 1.5 μl of cDNA sample or standard added to each well. The plate was covered with optically transparent sealing film and run on an Applied 7,500 Fast Real-Time PCR system. A melting curve was performed to validate the presence of single PCR product. Data was analysed on Applied Biosystem 7,500 Software 2.0.5 and the expression level of genes of interests were calculated using ΔCt method and normalized to GAPDH.

## Data Records

A project overview has been submitted as the BioProject reference PRJNA327913, with descriptions of the BioSample MDA-MB-231 D3H2LN Luc cells (reference SAMN07311741) and of the BioSample MDA-MB-435 cells (reference SAMN07311743). Unprocessed RNA-Sequencing reads have been deposited as fastq files at the National Center for Biotechnology Information (NCBI) Sequence Reads Archive (SRA) with the reference SRP111915 (Data Citation 1).

The fastq files correspond to four independent biological replicates (BiolRep1–4) for the MDA-MB-435 melanoma Parental, Selected1 or Selected2 cell populations, or to four independent biological replicates (BiolRep1–4) for the MDA-MB-231 Parental and three independent biological replicates (BiolRep1–3) for the Selected2, Selected3, FlowSorted1 or FlowSorted2 cell populations, as indicated in Table 2 (available online only). For each sequencing reaction run on the Illumina NextSeq 500 instrument, four technical replicates were produced (TechRep1–4). Forward (R1) and reverse (R2) reads have been combined, with SRA accession numbers for the combined sequencing results also indicated in Table 2 (available online only). Please also see the associated Metadata Record.

## Technical Validation

**Figure 3:**
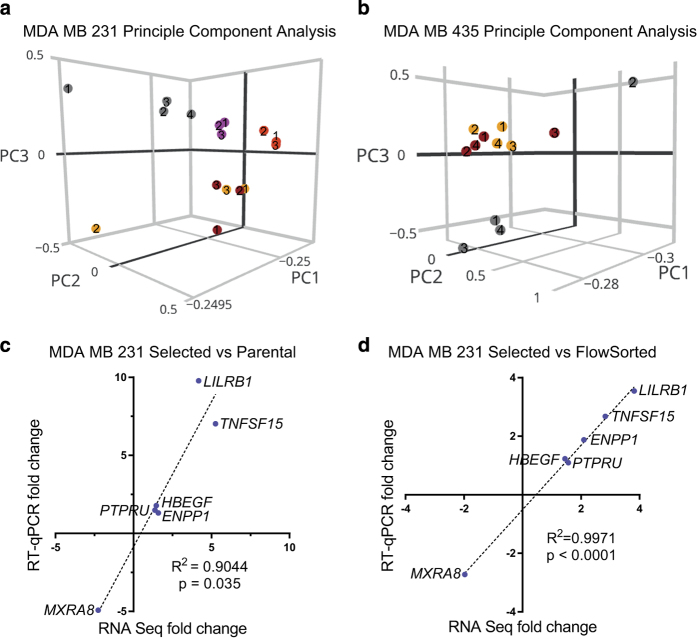
Quality control of RNA-Seq data. (**a**) Principal component plot of RNA-Seq (DESeq2) data from MDA-MB-231 Parental (grey symbols), Selected2 (orange symbol), Selected3 (brown symbol), FlowSorted1 (magenta symbol) and FlowSorted2 (red symbol) cell populations. Parental and FlowSorted populations clustered distinctly from Selected populations cluster. (**b**) Principal component plot of RNA-Seq (DESeq2) data from MDA-MB-435 Parental (grey symbols), Selected1 (brown symbol), and Selected2 (orange symbol) cell populations, with the number of the biological replicate indicated within each symbol. Parental cells clustered distinctly from the Selected populations cluster. (**c**) Linear regression plots of gene expression fold change (relative to *GAPDH*) for Selected MDA-MB-231 cells versus Parental or (**d**) versus FlowSorted cells obtained by RNA-Seq (DESeq2 data, relative to *GAPDH*) compared to RT-qPCR (relative to *GAPDH*). RNA transcripts expressed at relatively higher levels in Selected cells: *LILRB1*, *TNFSF15*, *HBEGF*, *PTPRU*, *ENPP1*. RNA transcript expressed at relatively lower levels in Selected cells: *MXRA8*. R^2^ of Goodness of Fit with *P* value.

The quality of extracted RNA (RNA integrity number equivalent; RINe) of all samples was determined using the Agilent RNA ScreenTape assay and the Agilent 2,200 TapeStation system (Table 3). Following RNA-seq, correlation coefficients were calculated for all pairwise comparisons of biological replicates (Table 5), which were ≥0.9874292 for MDA-MB-231 cells and ≥0.9995527 for MDA-MB-435 cells. Principal component analysis revealed that the four biological replicates of Parental MDA-MB-231 cells (Fig. 3a; grey symbols) along with the three biological replicates of FlowSorted1 and FlowSorted2 populations (Fig. 3a; magenta and red symbols) clustered together, while the three biological replicates of Selected2 and Selected3 populations (Fig. 3a; orange and brown symbols) formed a separate cluster. Similarly, three of the Parental MDA-MB-435 biological replicates (Fig. 3b; grey symbols) clustered together and were separate from the four biological replicates of Selected1 and Selected2 populations (Fig. 3b; orange and brown symbols). These results are consistent with the pore-selection procedure having enriched for independent cell populations with transcriptomic profiles distinct from the originating parental populations.

Further technical validation of the RNA-Sequencing results was provided by quantitative reverse transcription PCR (RT-qPCR) analyses of differences in gene expression between MDA-MB-231 Parental versus Selected (Fig. 3c) or MDA-MB-231 Selected versus FlowSorted cells (Fig. 3d) identified by RNA-Seq. Genes were selected on the basis of fold-change, statistical significance and number of sequence reads. The relatively higher expression of the genes encoding leukocyte immunoglobulin-like receptor subfamily B member 1 (*LILRB1*), tumour necrosis factor ligand superfamily member 15 (*TNFSF15*), ectonucleotide pyrophosphatase/phosphodiesterase 1 (*ENPP1*), protein tyrosine phosphatase receptor type U (*PTPRU*), heparin-binding epidermal growth factor (*HBEGF*), as well as the relatively lower expression of matrix remodelling associated 8 (*MXRA8*), in Selected versus Parental (Fig. 3c) as well as in Selected versus FlowSorted cells (Fig. 3d) were comparable in both RNA-Seq and RT-qPCR assays. In both cases, the fold-changes determined by either method fell on a single fitted straight line with R^2^>0.90 and *P*<0.05 (Fig. 3c,d). Although the agreement between RNA-Seq and RT-qPCR was good for this limited set of genes, it is formally possible that the analytical methods employed might have underestimated gene expression levels^15^.

## Additional Information

**How to cite this article:** Rudzka, D. A. *et al.* Transcriptomic profiling of human breast and melanoma cells selected by migration through narrow constraints. *Sci. Data* 4:170172 doi: 10.1038/sdata.2017.172 (2017).

**Publisher’s note:** Springer Nature remains neutral with regard to jurisdictional claims in published maps and institutional affiliations.

## Supplementary Material



## Figures and Tables

**Table 1:** Experimental samples and design.

**Cell line**	**BioSample**	**Cell population**	**Enrichment method**	**Biological replicates**	**Protocol 1**	**Protocol 2**
MDA-MB-231 D3H2LN Luc	SAMN07311741	Parental	None	4	RNA extraction	RNA-Seq
MDA-MB-231 D3H2LN Luc	SAMN07311741	Selected	Selection through 3 μm pores	3	RNA extraction	RNA-Seq
MDA-MB-231 D3H2LN Luc	SAMN07311741	FlowSorted	Selection for small diameter by flow cytometry	3	RNA extraction	RNA-Seq
MDA-MB-435	SAMN07311743	Parental	None	4	RNA extraction	RNA-Seq
MDA-MB-435	SAMN07311743	Selected	Selection through 3 μm pores	4	RNA extraction	RNA-Seq

**Table 2:** Description and names of data files deposited with SRA

**Cell line**	**Cell population**	**Biological replicate**	**Technical replicate**	**Total sequence reads**	**Data file ID**	**SRA accession**
MDA-MB-231 D3H2LN Luc	Parental	1	1	7.47E+06	MDAMB231_Parental_BiolRep1_TechRep1_R1.fastq.gz	SRR5826336
MDA-MB-231 D3H2LN Luc	Parental	1	2	7.42E+06	MDAMB231_Parental_BiolRep1_TechRep2_R1.fastq.gz	SRR5826335
MDA-MB-231 D3H2LN Luc	Parental	1	3	8.11E+06	MDAMB231_Parental_BiolRep1_TechRep3_R1.fastq.gz	SRR5826338
MDA-MB-231 D3H2LN Luc	Parental	1	4	8.00E+06	MDAMB231_Parental_BiolRep1_TechRep4_R1.fastq.gz	SRR5826337
MDA-MB-231 D3H2LN Luc	Parental	2	1	7.29E+06	MDAMB231_Parental_BiolRep2_TechRep1_R1.fastq.gz	SRR5826339
MDA-MB-231 D3H2LN Luc	Parental	2	2	7.23E+06	MDAMB231_Parental_BiolRep2_TechRep2_R1.fastq.gz	SRR5826341
MDA-MB-231 D3H2LN Luc	Parental	2	3	7.86E+06	MDAMB231_Parental_BiolRep2_TechRep3_R1.fastq.gz	SRR5826340
MDA-MB-231 D3H2LN Luc	Parental	2	4	7.77E+06	MDAMB231_Parental_BiolRep2_TechRep4_R1.fastq.gz	SRR5826343
MDA-MB-231 D3H2LN Luc	Parental	3	1	6.54E+06	MDAMB231_Parental_BiolRep3_TechRep1_R1.fastq.gz	SRR5826342
MDA-MB-231 D3H2LN Luc	Parental	3	2	6.45E+06	MDAMB231_Parental_BiolRep3_TechRep2_R1.fastq.gz	SRR5826344
MDA-MB-231 D3H2LN Luc	Parental	3	3	7.05E+06	MDAMB231_Parental_BiolRep3_TechRep3_R1.fastq.gz	SRR5826410
MDA-MB-231 D3H2LN Luc	Parental	3	4	6.95E+06	MDAMB231_Parental_BiolRep3_TechRep4_R1.fastq.gz	SRR5826411
MDA-MB-231 D3H2LN Luc	Parental	4	1	7.28E+06	MDAMB231_Parental_BiolRep4_TechRep1_R1.fastq.gz	SRR5826413
MDA-MB-231 D3H2LN Luc	Parental	4	2	7.23E+06	MDAMB231_Parental_BiolRep4_TechRep2_R1.fastq.gz	SRR5826412
MDA-MB-231 D3H2LN Luc	Parental	4	3	7.86E+06	MDAMB231_Parental_BiolRep4_TechRep3_R1.fastq.gz	SRR5826409
MDA-MB-231 D3H2LN Luc	Parental	4	4	7.77E+06	MDAMB231_Parental_BiolRep4_TechRep4_R1.fastq.gz	SRR5826424
MDA-MB-231 D3H2LN Luc	Selected2	1	1	7.17E+06	MDAMB231_Selected2_BiolRep1_TechRep1_R1.fastq.gz	SRR5826425
MDA-MB-231 D3H2LN Luc	Selected2	1	2	7.10E+06	MDAMB231_Selected2_BiolRep1_TechRep2_R1.fastq.gz	SRR5826422
MDA-MB-231 D3H2LN Luc	Selected2	1	3	7.77E+06	MDAMB231_Selected2_BiolRep1_TechRep3_R1.fastq.gz	SRR5826423
MDA-MB-231 D3H2LN Luc	Selected2	1	4	7.65E+06	MDAMB231_Selected2_BiolRep1_TechRep4_R1.fastq.gz	SRR5826427
MDA-MB-231 D3H2LN Luc	Selected2	2	1	8.19E+06	MDAMB231_Selected2_BiolRep2_TechRep1_R1.fastq.gz	SRR5826388
MDA-MB-231 D3H2LN Luc	Selected2	2	2	8.13E+06	MDAMB231_Selected2_BiolRep2_TechRep2_R1.fastq.gz	SRR5826389
MDA-MB-231 D3H2LN Luc	Selected2	2	3	8.84E+06	MDAMB231_Selected2_BiolRep2_TechRep3_R1.fastq.gz	SRR5826386
MDA-MB-231 D3H2LN Luc	Selected2	2	4	8.73E+06	MDAMB231_Selected2_BiolRep2_TechRep4_R1.fastq.gz	SRR5826387
MDA-MB-231 D3H2LN Luc	Selected2	3	1	7.18E+06	MDAMB231_Selected2_BiolRep3_TechRep1_R1.fastq.gz	SRR5826385
MDA-MB-231 D3H2LN Luc	Selected2	3	2	7.10E+06	MDAMB231_Selected2_BiolRep3_TechRep2_R1.fastq.gz	SRR5826401
MDA-MB-231 D3H2LN Luc	Selected2	3	3	7.75E+06	MDAMB231_Selected2_BiolRep3_TechRep3_R1.fastq.gz	SRR5826400
MDA-MB-231 D3H2LN Luc	Selected2	3	4	7.64E+06	MDAMB231_Selected2_BiolRep3_TechRep4_R1.fastq.gz	SRR5826399
MDA-MB-231 D3H2LN Luc	Selected3	1	1	7.69E+06	MDAMB231_Selected3_BiolRep1_TechRep1_R1.fastq.gz	SRR5826398
MDA-MB-231 D3H2LN Luc	Selected3	1	2	7.63E+06	MDAMB231_Selected3_BiolRep1_TechRep2_R1.fastq.gz	SRR5826397
MDA-MB-231 D3H2LN Luc	Selected3	1	3	8.29E+06	MDAMB231_Selected3_BiolRep1_TechRep3_R1.fastq.gz	SRR5826367
MDA-MB-231 D3H2LN Luc	Selected3	1	4	8.19E+06	MDAMB231_Selected3_BiolRep1_TechRep4_R1.fastq.gz	SRR5826407
MDA-MB-231 D3H2LN Luc	Selected3	2	1	6.72E+06	MDAMB231_Selected3_BiolRep2_TechRep1_R1.fastq.gz	SRR5826359
MDA-MB-231 D3H2LN Luc	Selected3	2	2	6.66E+06	MDAMB231_Selected3_BiolRep2_TechRep2_R1.fastq.gz	SRR5826360
MDA-MB-231 D3H2LN Luc	Selected3	2	3	7.32E+06	MDAMB231_Selected3_BiolRep2_TechRep3_R1.fastq.gz	SRR5826368
MDA-MB-231 D3H2LN Luc	Selected3	2	4	7.20E+06	MDAMB231_Selected3_BiolRep2_TechRep4_R1.fastq.gz	SRR5826375
MDA-MB-231 D3H2LN Luc	Selected3	3	1	7.02E+06	MDAMB231_Selected3_BiolRep3_TechRep1_R1.fastq.gz	SRR5826374
MDA-MB-231 D3H2LN Luc	Selected3	3	2	6.96E+06	MDAMB231_Selected3_BiolRep3_TechRep2_R1.fastq.gz	SRR5826376
MDA-MB-231 D3H2LN Luc	Selected3	3	3	7.59E+06	MDAMB231_Selected3_BiolRep3_TechRep3_R1.fastq.gz	SRR5826377
MDA-MB-231 D3H2LN Luc	Selected3	3	4	7.48E+06	MDAMB231_Selected3_BiolRep3_TechRep4_R1.fastq.gz	SRR5826378
MDA-MB-231 D3H2LN Luc	FlowSorted1	1	1	7.38E+06	MDAMB231_FlowSorted1_BiolRep1_TechRep1_R1.fastq.gz	SRR5826437
MDA-MB-231 D3H2LN Luc	FlowSorted1	1	2	7.19E+06	MDAMB231_FlowSorted1_BiolRep1_TechRep2_R1.fastq.gz	SRR5826438
MDA-MB-231 D3H2LN Luc	FlowSorted1	1	3	7.96E+06	MDAMB231_FlowSorted1_BiolRep1_TechRep3_R1.fastq.gz	SRR5826439
MDA-MB-231 D3H2LN Luc	FlowSorted1	1	4	7.74E+06	MDAMB231_FlowSorted1_BiolRep1_TechRep4_R1.fastq.gz	SRR5826426
MDA-MB-231 D3H2LN Luc	FlowSorted1	2	1	7.46E+06	MDAMB231_FlowSorted1_BiolRep2_TechRep1_R1.fastq.gz	SRR5826408
MDA-MB-231 D3H2LN Luc	FlowSorted1	2	2	7.38E+06	MDAMB231_FlowSorted1_BiolRep2_TechRep2_R1.fastq.gz	SRR5826361
MDA-MB-231 D3H2LN Luc	FlowSorted1	2	3	8.07E+06	MDAMB231_FlowSorted1_BiolRep2_TechRep3_R1.fastq.gz	SRR5826349
MDA-MB-231 D3H2LN Luc	FlowSorted1	2	4	7.96E+06	MDAMB231_FlowSorted1_BiolRep2_TechRep4_R1.fastq.gz	SRR5826352
MDA-MB-231 D3H2LN Luc	FlowSorted1	3	1	7.63E+06	MDAMB231_FlowSorted1_BiolRep3_TechRep1_R1.fastq.gz	SRR5826351
MDA-MB-231 D3H2LN Luc	FlowSorted1	3	2	7.56E+06	MDAMB231_FlowSorted1_BiolRep3_TechRep2_R1.fastq.gz	SRR5826384
MDA-MB-231 D3H2LN Luc	FlowSorted1	3	3	8.23E+06	MDAMB231_FlowSorted1_BiolRep3_TechRep3_R1.fastq.gz	SRR5826362
MDA-MB-231 D3H2LN Luc	FlowSorted1	3	4	8.13E+06	MDAMB231_FlowSorted1_BiolRep3_TechRep4_R1.fastq.gz	SRR5826363
MDA-MB-231 D3H2LN Luc	FlowSorted2	1	1	7.23E+06	MDAMB231_FlowSorted2_BiolRep1_TechRep1_R1.fastq.gz	SRR5826364
MDA-MB-231 D3H2LN Luc	FlowSorted2	1	2	7.20E+06	MDAMB231_FlowSorted2_BiolRep1_TechRep2_R1.fastq.gz	SRR5826365
MDA-MB-231 D3H2LN Luc	FlowSorted2	1	3	7.84E+06	MDAMB231_FlowSorted2_BiolRep1_TechRep3_R1.fastq.gz	SRR5826366
MDA-MB-231 D3H2LN Luc	FlowSorted2	1	4	7.76E+06	MDAMB231_FlowSorted2_BiolRep1_TechRep4_R1.fastq.gz	SRR5826346
MDA-MB-231 D3H2LN Luc	FlowSorted2	2	1	6.93E+06	MDAMB231_FlowSorted2_BiolRep2_TechRep1_R1.fastq.gz	SRR5826345
MDA-MB-231 D3H2LN Luc	FlowSorted2	2	2	6.89E+06	MDAMB231_FlowSorted2_BiolRep2_TechRep2_R1.fastq.gz	SRR5826348
MDA-MB-231 D3H2LN Luc	FlowSorted2	2	3	7.48E+06	MDAMB231_FlowSorted2_BiolRep2_TechRep3_R1.fastq.gz	SRR5826347
MDA-MB-231 D3H2LN Luc	FlowSorted2	2	4	7.40E+06	MDAMB231_FlowSorted2_BiolRep2_TechRep4_R1.fastq.gz	SRR5826350
MDA-MB-231 D3H2LN Luc	FlowSorted2	3	1	6.49E+06	MDAMB231_FlowSorted2_BiolRep3_TechRep1_R1.fastq.gz	SRR5826436
MDA-MB-231 D3H2LN Luc	FlowSorted2	3	2	6.47E+06	MDAMB231_FlowSorted2_BiolRep3_TechRep2_R1.fastq.gz	SRR5826435
MDA-MB-231 D3H2LN Luc	FlowSorted2	3	3	7.05E+06	MDAMB231_FlowSorted2_BiolRep3_TechRep3_R1.fastq.gz	SRR5826433
MDA-MB-231 D3H2LN Luc	FlowSorted2	3	4	6.97E+06	MDAMB231_FlowSorted2_BiolRep3_TechRep4_R1.fastq.gz	SRR5826434
MDA-MB-435	Parental	1	1	1.11E+07	MDAMB435_Parental_BiolRep1_TechRep1_R1.fastq.gz	SRR5826440
MDA-MB-435	Parental	1	2	1.08E+07	MDAMB435_Parental_BiolRep1_TechRep2_R1.fastq.gz	SRR5826429
MDA-MB-435	Parental	1	3	1.17E+07	MDAMB435_Parental_BiolRep1_TechRep3_R1.fastq.gz	SRR5826430
MDA-MB-435	Parental	1	4	1.13E+07	MDAMB435_Parental_BiolRep1_TechRep4_R1.fastq.gz	SRR5826431
MDA-MB-435	Parental	2	1	1.16E+07	MDAMB435_Parental_BiolRep2_TechRep1_R1.fastq.gz	SRR5826432
MDA-MB-435	Parental	2	2	1.12E+07	MDAMB435_Parental_BiolRep2_TechRep2_R1.fastq.gz	SRR5826428
MDA-MB-435	Parental	2	3	1.21E+07	MDAMB435_Parental_BiolRep2_TechRep3_R1.fastq.gz	SRR5826418
MDA-MB-435	Parental	2	4	1.18E+07	MDAMB435_Parental_BiolRep2_TechRep4_R1.fastq.gz	SRR5826419
MDA-MB-435	Parental	3	1	1.06E+07	MDAMB435_Parental_BiolRep3_TechRep1_R1.fastq.gz	SRR5826416
MDA-MB-435	Parental	3	2	1.02E+07	MDAMB435_Parental_BiolRep3_TechRep2_R1.fastq.gz	SRR5826417
MDA-MB-435	Parental	3	3	1.11E+07	MDAMB435_Parental_BiolRep3_TechRep3_R1.fastq.gz	SRR5826414
MDA-MB-435	Parental	3	4	1.08E+07	MDAMB435_Parental_BiolRep3_TechRep4_R1.fastq.gz	SRR5826406
MDA-MB-435	Parental	4	1	1.33E+07	MDAMB435_Parental_BiolRep4_TechRep1_R1.fastq.gz	SRR5826405
MDA-MB-435	Parental	4	2	1.29E+07	MDAMB435_Parental_BiolRep4_TechRep2_R1.fastq.gz	SRR5826404
MDA-MB-435	Parental	4	3	1.40E+07	MDAMB435_Parental_BiolRep4_TechRep3_R1.fastq.gz	SRR5826403
MDA-MB-435	Parental	4	4	1.36E+07	MDAMB435_Parental_BiolRep4_TechRep4_R1.fastq.gz	SRR5826402
MDA-MB-435	Selected1	1	1	1.21E+07	MDAMB435_Selected1_BiolRep1_TechRep1_R1.fastq.gz	SRR5826391
MDA-MB-435	Selected1	1	2	1.17E+07	MDAMB435_Selected1_BiolRep1_TechRep2_R1.fastq.gz	SRR5826390
MDA-MB-435	Selected1	1	3	1.27E+07	MDAMB435_Selected1_BiolRep1_TechRep3_R1.fastq.gz	SRR5826393
MDA-MB-435	Selected1	1	4	1.23E+07	MDAMB435_Selected1_BiolRep1_TechRep4_R1.fastq.gz	SRR5826392
MDA-MB-435	Selected1	2	1	1.35E+07	MDAMB435_Selected1_BiolRep2_TechRep1_R1.fastq.gz	SRR5826394
MDA-MB-435	Selected1	2	2	1.31E+07	MDAMB435_Selected1_BiolRep2_TechRep2_R1.fastq.gz	SRR5826381
MDA-MB-435	Selected1	2	3	1.42E+07	MDAMB435_Selected1_BiolRep2_TechRep3_R1.fastq.gz	SRR5826382
MDA-MB-435	Selected1	2	4	1.37E+07	MDAMB435_Selected1_BiolRep2_TechRep4_R1.fastq.gz	SRR5826379
MDA-MB-435	Selected1	3	1	1.19E+07	MDAMB435_Selected1_BiolRep3_TechRep1_R1.fastq.gz	SRR5826380
MDA-MB-435	Selected1	3	2	1.16E+07	MDAMB435_Selected1_BiolRep3_TechRep2_R1.fastq.gz	SRR5826383
MDA-MB-435	Selected1	3	3	1.25E+07	MDAMB435_Selected1_BiolRep3_TechRep3_R1.fastq.gz	SRR5826370
MDA-MB-435	Selected1	3	4	1.21E+07	MDAMB435_Selected1_BiolRep3_TechRep4_R1.fastq.gz	SRR5826371
MDA-MB-435	Selected1	4	1	1.12E+07	MDAMB435_Selected1_BiolRep4_TechRep1_R1.fastq.gz	SRR5826372
MDA-MB-435	Selected1	4	2	1.08E+07	MDAMB435_Selected1_BiolRep4_TechRep2_R1.fastq.gz	SRR5826373
MDA-MB-435	Selected1	4	3	1.17E+07	MDAMB435_Selected1_BiolRep4_TechRep3_R1.fastq.gz	SRR5826369
MDA-MB-435	Selected1	4	4	1.14E+07	MDAMB435_Selected1_BiolRep4_TechRep4_R1.fastq.gz	SRR5826356
MDA-MB-435	Selected2	1	1	1.17E+07	MDAMB435_Selected2_BiolRep1_TechRep1_R1.fastq.gz	SRR5826355
MDA-MB-435	Selected2	1	2	1.13E+07	MDAMB435_Selected2_BiolRep1_TechRep2_R1.fastq.gz	SRR5826358
MDA-MB-435	Selected2	1	3	1.23E+07	MDAMB435_Selected2_BiolRep1_TechRep3_R1.fastq.gz	SRR5826357
MDA-MB-435	Selected2	1	4	1.19E+07	MDAMB435_Selected2_BiolRep1_TechRep4_R1.fastq.gz	SRR5826354
MDA-MB-435	Selected2	2	1	1.25E+07	MDAMB435_Selected2_BiolRep2_TechRep1_R1.fastq.gz	SRR5826444
MDA-MB-435	Selected2	2	2	1.20E+07	MDAMB435_Selected2_BiolRep2_TechRep2_R1.fastq.gz	SRR5826443
MDA-MB-435	Selected2	2	3	1.31E+07	MDAMB435_Selected2_BiolRep2_TechRep3_R1.fastq.gz	SRR5826446
MDA-MB-435	Selected2	2	4	1.27E+07	MDAMB435_Selected2_BiolRep2_TechRep4_R1.fastq.gz	SRR5826445
MDA-MB-435	Selected2	3	1	1.16E+07	MDAMB435_Selected2_BiolRep3_TechRep1_R1.fastq.gz	SRR5826441
MDA-MB-435	Selected2	3	2	1.12E+07	MDAMB435_Selected2_BiolRep3_TechRep2_R1.fastq.gz	SRR5826442
MDA-MB-435	Selected2	3	3	1.21E+07	MDAMB435_Selected2_BiolRep3_TechRep3_R1.fastq.gz	SRR5826415
MDA-MB-435	Selected2	3	4	1.18E+07	MDAMB435_Selected2_BiolRep3_TechRep4_R1.fastq.gz	SRR5826420
MDA-MB-435	Selected2	4	1	1.20E+07	MDAMB435_Selected2_BiolRep4_TechRep1_R1.fastq.gz	SRR5826421
MDA-MB-435	Selected2	4	2	1.16E+07	MDAMB435_Selected2_BiolRep4_TechRep2_R1.fastq.gz	SRR5826353
MDA-MB-435	Selected2	4	3	1.26E+07	MDAMB435_Selected2_BiolRep4_TechRep3_R1.fastq.gz	SRR5826395
MDA-MB-435	Selected2	4	4	1.22E+07	MDAMB435_Selected2_BiolRep4_TechRep4_R1.fastq.gz	SRR5826396

**Table 3:** RNA integrity values for each sample used for RNA sequencing.

**MDA-MB-231**		**MDA-MB-435**	
**Sample name**	**RINe**	**Sample name**	**RINe**
Parental1	10.0	Parental1	10.0
Parental2	9.9	Parental2	10.0
Parental3	10.0	Parental3	10.0
Parental4	9.9	Parental4	10.0
Selected2-1	10.0	Selected1-1	10.0
Selected2-2	9.9	Selected1-2	10.0
Selected2-3	10.0	Selected1-3	10.0
Selected3-1	10.0	Selected1-4	9.6
Selected3-2	8.9	Selected2-1	10.0
Selected3-3	9.9	Selected2-2	10.0
FlowSorted1-1	10.0	Selected2-3	10.0
FlowSorted1-2	10.0	Selected2-4	10.0
FlowSorted1-3	10.0		
FlowSorted2-1	9.9		
FlowSorted2-2	10.0		
FlowSorted2-3	10.0		

**Table 4:** PCR primer catalogue numbers.

**Primer target**	**Qiagen catalogue number**
*LILRB1*	QT00024920
*TNFSF15*	QT00041965
*HBEGF*	QT00000455
*PTPRU*	QT00005677
*ENPP1*	QT00094787
*MXRA8*	QT00202790
*GAPDH*	QT00079247

**Table 5:** Pearson’s correlation coefficient (r) values for biological replicates.

**MDA-MB-231**			**MDA-MB-435**		
**Replicate**	**versus Replicate**	**Pearson’s r**	**Replicate**	**versus Replicate**	**Pearson’s r**
Parental1	Parental2	0.9960374	Parental1	Parental2	0.9996197
Parental1	Parental3	0.9965765	Parental1	Parental3	0.9999907
Parental1	Parental4	0.9893210	Parental1	Parental4	0.9999899
Parental2	Parental3	0.9972366	Parental2	Parental3	0.9995527
Parental2	Parental4	0.9951007	Parental2	Parental4	0.9996528
Parental3	Parental4	0.9954966	Parental3	Parental4	0.9999925
Selected2-1	Selected2-2	0.9879274	Selected1-1	Selected1-2	0.9999951
Selected2-1	Selected2-3	0.9949912	Selected1-1	Selected1-3	0.9999855
Selected2-2	Selected2-3	0.9924361	Selected1-1	Selected1-4	0.9999968
Selected3-1	Selected3-2	0.9979480	Selected1-2	Selected1-3	0.9999798
Selected3-1	Selected3-3	0.9968270	Selected1-2	Selected1-4	0.9999967
Selected3-2	Selected3-3	0.9974801	Selected1-3	Selected1-4	0.9999865
FlowSorted1-1	FlowSorted1-2	0.9955907	Selected2-1	Selected2-2	0.9999973
FlowSorted1-1	FlowSorted1-3	0.9969312	Selected2-1	Selected2-3	0.9999610
FlowSorted1-2	FlowSorted1-3	0.9967215	Selected2-1	Selected2-4	0.9999835
FlowSorted2-1	FlowSorted2-2	0.9874292	Selected2-2	Selected2-3	0.9999461
FlowSorted2-1	FlowSorted2-3	0.9990905	Selected2-2	Selected2-4	0.9999739
FlowSorted2-2	FlowSorted2-3	0.9913348	Selected2-3	Selected2-4	0.9999938
